# Band keratopathy

**DOI:** 10.5935/0004-2749.2025-0315

**Published:** 2025-11-24

**Authors:** João Vitor Laranjeira, Nicole Bulgarão Maricondi de Almeida, Newton Kara-Junior

**Affiliations:** 1 Ophthalmology department, Hospital das Clínicas, Universidade de São Paulo, São Paulo, SP, Brazil

Band keratopathy (Figure) is a degenerative disease localized in the interpalpebral aperture^(1)^. It consists of band-shaped fine calcium deposits in the anterior corneal layers^(2)^. Its mechanism is unknown^(1)^. Early stages are asymptomatic^(2)^; however, over time, common complaints include a sensation of a foreign body in the eye, lachrymation, photophobia, visual acuity deterioration, and glare^(1)^. Treatment aims to restore corneal surface, including mechanical debridement, superficial keratectomy, ethylenediaminetetraacetic acid chelation, and phototherapeutic keratectomy^(2)^.

**Figure:**
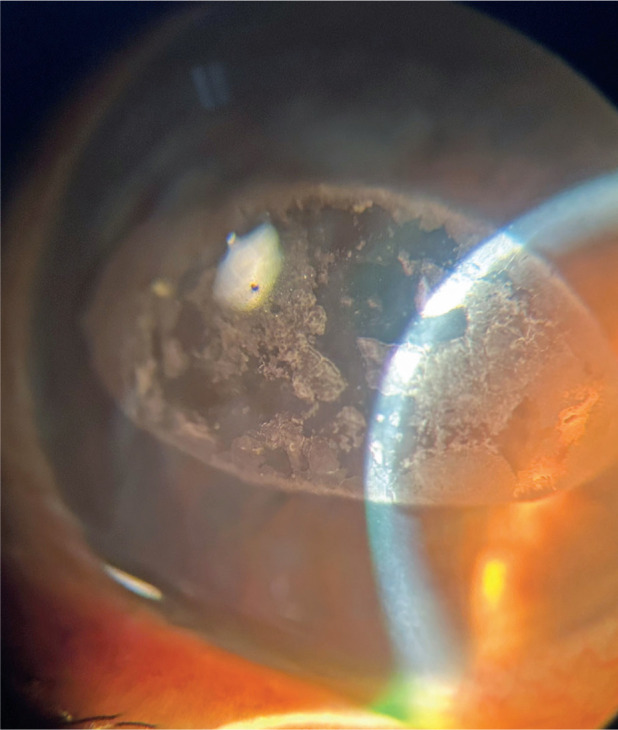
Band keratopathy.

## Data Availability

The datasets generated and/or analyzed during the current study are included in the manuscript.
